# Identifying Molecular-Based Trophic Interactions as a Resource for Advanced Integrated Pest Management

**DOI:** 10.3390/insects12040358

**Published:** 2021-04-16

**Authors:** Jason M. Schmidt, Angelita Acebes-Doria, Brett Blaauw, Arash Kheirodin, Swikriti Pandey, Kylie Lennon, Amos D. Kaldor, Pedro F. S. Toledo, Erin E. Grabarczyk

**Affiliations:** 1 Department of Entomology, Tifton Campus, University of Georgia, Tifton, GA 31794, USA; aacebes@uga.edu (A.A.-D.); Arash.Kheirodin@uga.edu (A.K.); Swikriti.Pandey@uga.edu (S.P.); Kylie.Lennon@uga.edu (K.L.); toledo@uga.edu (P.F.S.T.); 2 Department of Entomology, Athens Campus, University of Georgia, Athens, GA 30602, USA; bblaauw@uga.edu (B.B.); Amos.Kaldor@uga.edu (A.D.K.); 3 Southeast Watershed Research, USDA, Tifton, GA 31793, USA; Erin.Grabarczyk@usda.gov

**Keywords:** agricultural communities, ELISA, food-webs, gut content analysis, metabarcoding, molecular trophic interactions, NGS, PCR, species interactions

## Abstract

**Simple Summary:**

With increasing human populations and the need for ecosystem services to work in synergy with the production of specialty crops, the maintenance of biodiversity is becoming increasingly important. The aims of this study were to review the current literature employing molecular analysis to reveal the roles of species in providing biological control in agricultural systems. Decrypting the trophic networks between biological control agents and agricultural pests is essential to build eco-friendly strategies that promote the natural management of pests before any mediations, such as chemical control strategies, are required. It was found, during the review process, that our understanding of biological control communities is lacking in many agricultural systems, including common fruit and vegetable production, both in terms of what species are doing for crop production, and how various environmental challenges (i.e., land-use and habitat management concepts, such as wildflower borders) influence species interactions and the delivery of biological control services. New techniques harvesting the power of DNA to reveal species’ roles in specialty crops are an avenue forward to help integrate natural pest management into our standard operating procedures.

**Abstract:**

Biodiversity is an essential attribute of sustainable agroecosystems. Diverse arthropod communities deliver multiple ecosystem services, such as biological control, which are the core of integrated pest management programs. The molecular analysis of arthropod diets has emerged as a new tool to monitor and help predict the outcomes of management on the functioning of arthropod communities. Here, we briefly review the recent molecular analysis of predators and parasitoids in agricultural environments. We focus on the developments of molecular gut content analysis (MGCA) implemented to unravel the function of community members, and their roles in biological control. We examine the agricultural systems in which this tool has been applied, and at what ecological scales. Additionally, we review the use of MGCA to uncover vertebrate roles in pest management, which commonly receives less attention. Applying MGCA to understand agricultural food webs is likely to provide an indicator of how management strategies either improve food web properties (i.e., enhanced biological control), or adversely impact them.

## 1. Introduction

Biological control, or pest control by living organisms, is a central component of integrated pest management [1]. In the US alone, biological control services are valued in the billions of dollars [2]). A body of research has established that promoting the diversity of biocontrol agents significantly contributes to crop production around the world [3,4,5]. Therefore, maintaining species diversity within agroecosystems is essential for sustainability. In order to preserve production, we must advance our current integration of biodiversity into common agricultural practice [3]. In order to better understand the outcomes of biodiversity, the molecular analysis of trophic interactions is a tool that can be used to elucidate species’ ecological roles and resource use in agroecosystems. 

Species identity in predator–prey interactions is important, but understanding the functional role that predators fulfill within a focal community better illustrates patterns of biological control across systems. Therefore, a shift towards promoting diverse communities in terms of species roles and the distribution of ‘functional’ traits will move our understanding of biological control forward for specialty cropping systems [6]. For example, the efficacy of biological control by predatory arthropods depends in part on feeding specialization [7]. Diverse communities should, therefore, be composed of a variety of functional traits, such as differences in size, feeding specialization, dispersal ability, activity, and hunting mode, etc. [6,8]. However, for most predatory species, we know little about their functional roles in terms of feeding on other arthropods and pests. Furthermore, the ways in which the functional traits of food webs are altered by management and how the structure of food webs enhances pest management remains poorly known [6]. 

The molecular analysis of arthropods and their associated interactions has emerged as a diagnostic approach to reveal animal diets and foraging traits [9]. The molecular diagnostics of the predator diets or parasitoids of pests can be accomplished by a variety of tools, such as antibody tests, protein markers, polymerase chain reactions (PCR), and DNA sequencing [1,13,14]. One benefit of characterizing predator diets with molecular diagnostics is the ability to estimate the natural predation within the environment and without the manipulation of potential interactions [15]. Moreover, with sequencing advances, the potential for the quantitative assessment of food web properties is possible i.e., connectivity and specialization [16,17]. Although limitations and challenges exist, particularly in terms of quantifying the rate of predation [9], molecular gut content analysis (hereafter MGCA) is a powerful approach which documents the predators of key pests and elucidates predator diets to reconstruct food webs. Here, we review the current literature which implements MGCA in agricultural systems, and we illustrate the power of MGCA in agricultural research to understand the role of predators in biological control programs [18]. We focus on studies from between 2015 and early 2020 to provide: (1) an overview of MGCA in agricultural systems, and (2) to highlight approaches used; (3) implications of research for biocontrol and IPM, and (4) to propose future research directions based on gaps in knowledge identified through the review process. A complete detailing of the methods and process of MGCA is beyond the scope of this review. We provide a brief description of the key terminology: see Box 1. In basic terms, the process of the molecular diagnostics of feeding links is the collection samples of fecal material, portions of animal guts, or whole individuals, and the application of a variety of currently-available molecular methods to screen sample material for prey remains. For additional information not included in this review, we refer readers to [1,9,13,14,19] for an overview of molecular gut content analysis, and King et al. [20] for techniques and best practices.

Box 1 **Some abbreviations and terms used in describing molecular trophic interactions.**

**MGCA**—Molecular gut content analysis is the process of using florescent dye, protein marker or DNA based tools to detect prey remains with the gut contents of predators.
   
**ELISA**—Enzyme-linked immunosorbent assay, which is a protein detection system de-signed to mark prey or detect specific proteins within the gut contents of a predator (e.g., Hagler and Durand 1994).
   
**PCR or Diagnostic PCR**—Polymerase chain reaction. Technique used to create copies of target DNA and in this application, used for diagnostic assessment of prey recently consumed by predators [9]. Or, for detecting parasitoids within hosts [10].
   
**NGS**—Next generation sequencing. Massively parallel sequencing technology capable of producing millions of sequences of DNA or RNA in mixed samples.
   
**DNA metabarcoding or molecular barcoding**—An application of NGS where commonly 6–8 bp MID tags (molecular identification tags or codes) are added to the PCR primers to mark each sample and allows for tracking samples that are combined to form libraries of samples that are then run together. Sequences, reads recovered from samples, are later used to determine the species in samples by the MID tags for each sample to assemble trophic links using available sequence data in GenBank and BOLD [11].
   
**eDNA**—Environmental DNA from any sample source. These could be from soil or in-sect or plant, pollen on legs of bees, arthropod exuviae, swabs of external body, or whole body. eDNA can then be used to reconstruct trophic interactions using NGS techniques such as DNA metabarcoding e.g., [12].
   
**HTS**—High-throughput sequencing techniques using NGS to power the rapid processing of thousands of samples simultaneously to explore biodiversity or trophic structure in agricultural systems.

## 2. Methods: The Review Process and Scope

**Figure 1:**
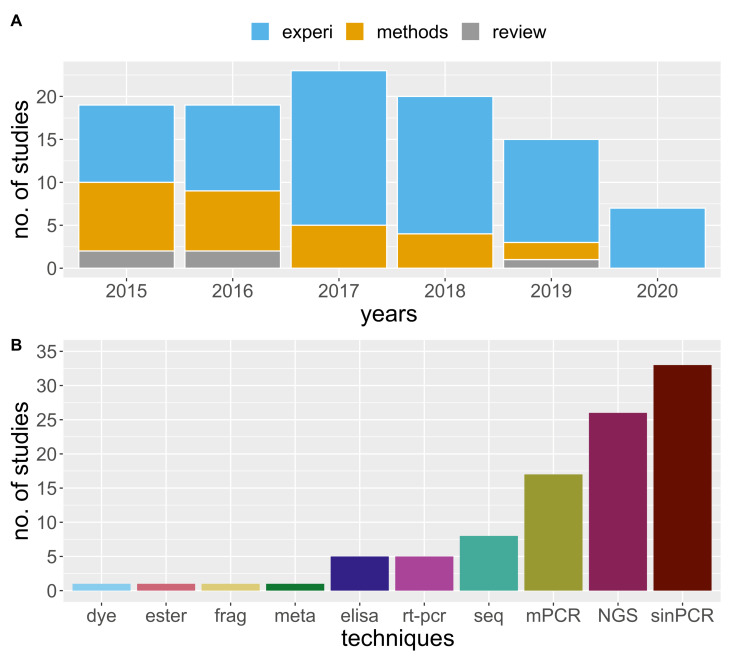
Distribution of molecular trophic interaction studies between 2015 and June 2020 by study type: “experi” for experimental, “methods” for development of new primers or optimization, and “review” for articles that reviewed molecular topics (**A**), and by the technique used to identify interaction (**B**). The technique codes are: fluorescent dye for animals marked with dye, ester for esterase, fragment analysis for frag, meta for metabaroding, elisa for Enzyme-Linked Immunosorbent Assay, rt-pcr for real time or quantitative pcr, seq for sanger sequencing, mPCR for multiplex PCR, NGS for next generation sequencing, and sinPCR for singleplex PCR.

**Figure 2:**
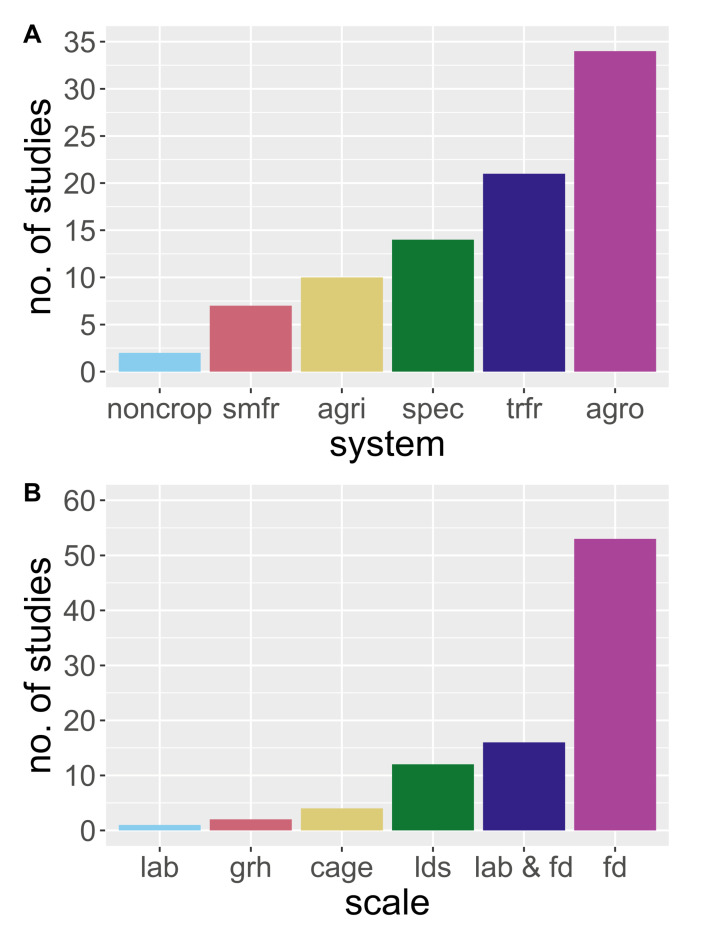
Distribution of molecular trophic interactions in agricultural systems by system (**A**), and the scale at which the study was conducted (**B**). The abbreviations in (**A**) indicate: noncrop = non crop habitat, smfr = small fruit, agri = agricultural landscapes, spec = specialty crops, trfr = tree fruit, and agro = agronomic crops. The abbreviations in (**B**) indicate: lab = laboratory study, grh = greenhouse study, cage = cage study, lds = landscape study, lab&fd = laboratory study combined with field study.

**Figure 3:**
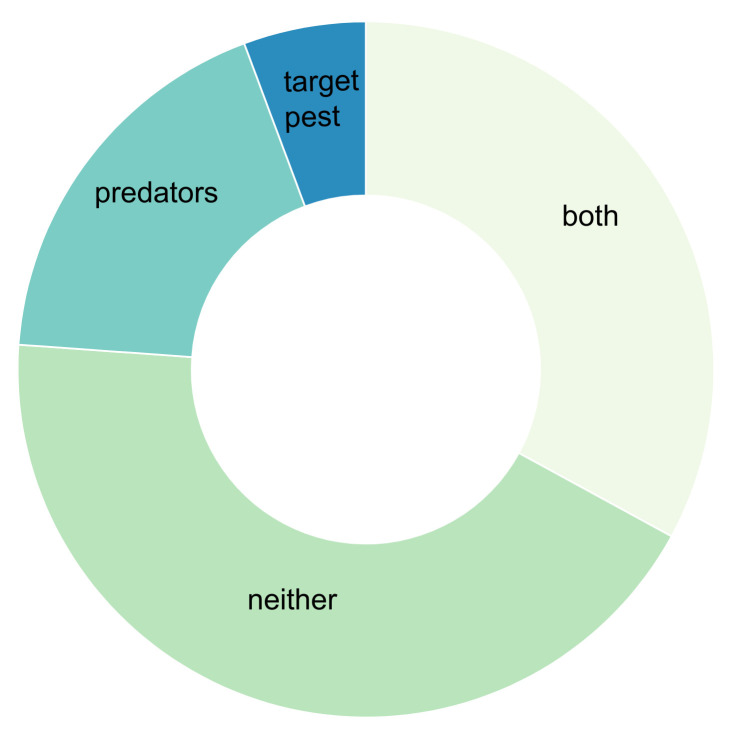
Distribution of molecular-based trophic interaction studies by whether the studies included estimates of abundance for pests or predators.

Following the call for MGCA research in agricultural systems outlined by González-Chang, Wratten, Lefort and Boyer [14], we used the same search criteria to isolate research published between 2015 and June 2020. We provide an updated review [14] regarding the body of knowledge pertaining to the use of molecular gut content analysis in order to understand agricultural food webs. We used Web of Science and Google Scholar search engines, and our process followed González-Chang, Wratten, Lefort and Boyer [14], omitting research not related to agriculture. During our review process, we added research on vertebrate predators that have long been considered of potential biological control significance (i.e., we removed the NOT “vertebrate”, NOT “bats”, NOT “bird*” from [11] the search criteria). In total, we found 104 qualifying studies, and during our search tallied the number of studies by: (1) the content (empirical, methodology development or review by year; “empiri”, “methods”, “review”; Figure 1A), (2) the techniques employed (Figure 1B), (3) the system of study (Figure 2A), and (4) the scale of the study (Figure 2B). Although there are many recent publications regarding the development of the methodology, we limited the inclusion of such studies to those focused specifically on agricultural systems. The type of data collected in association with molecular data matters; reporting additional data, such as abundance, in addition to trophic linkages using MGCA techniques will further our understanding of the interactions that occur in agroecosystems [19]. The benefit of combining abundance data with the occurrence of prey in predator guts, or the occurrence of different hosts in herbivores, is the ability to subsequently use network analysis to model how a change in abundance or interactions may alter the system’s function [17]. Therefore, as part of our review, we recorded the number of studies that included the abundance data of the predators of interest, prey, or both (Figure 3).

We found a constant publication rate of studies that employ MGCA in agricultural systems between 2015 and 2020 (Figure 1A; note partial year for 2020). Single PCR on a single pest was the primary technique used to diagnose molecular trophic interactions. However, since 2015, multiplex PCR (mPCR) and NGS analysis have become more common. MGCA was used in over 50 crops, and for this review, we combined these crops into common agricultural systems, and found that most studies were conducted in agronomic crops. The molecular analysis of trophic interactions is a powerful approach which reveals how ecological communities function in agricultural landscapes [14]. However, few studies were devoted specifically to non-crop habitat or agricultural habitats in general; most are limited to field scales, small plots, or with combined field and laboratory approaches (Figure 2B). Below, we review examples and outputs of research conducted within specific agricultural systems, highlighted in Figure 2A. We emphasize the use of MGCA to determine ecosystem disservices within systems—such as intraguild predation, the need to work in specialty crops, and agricultural landscapes—and end by reviewing promising new research regarding the role of vertebrate biocontrol services.

## 3. Results

### 3.1. MGCA in Agronomic Cropping Systems

Most of the recent studies of MGCA focused on agronomic crops, and approximately half were conducted in cereal systems (Figure 2A). Many studies targeted one pest using PCR, and the remaining used a mixture of multiplex and sequencing. The most comprehensive studies used multiplex PCR coupled with modeling to predict trophic structure and network parameters [21,22,23,24,25] and pest host-plant diet breadth [26,27], or explored the effects of cropping systems on predator–prey interactions [28,29]. In this section, we provide an overview of the results from the application of MGCA to study predator–pest interactions in agronomic crop systems.

Many recent MGCA studies in agronomic crops have sought to understand the role of generalist arthropod predators, which are abundant in agroecosystems. MGCA is often used to assess the efficacy of predators as potential biocontrol agents for a targeted pest. For example, *Orius insidiosus* (Hemiptera: Anthocoridae; Say), a generalist predator observed in corn, was identified as an effective biological control agent for the management of a serious pest, *Helicoverpa zea* (Leptidoptera: Noctuidae; Boddie) [30]. Excitingly, conservation practices elevated predation levels on *H. zea* [30]. Predators may be unevenly distributed throughout a system; Kheirodin et al. [31] determined that the predation of the cereal leaf beetle by foliar predators was higher compared to ground-dwelling species, suggesting that conservation should focus on improving foliar predator numbers. The PCR-based gut content analysis used to examine stink bug predation revealed that a diversity of arthropod predators from adjoining cotton–soybean fields tested positive for stink bug DNA [21]. MGCA can also reveal that generalist predators may rarely test positive for feeding on a targeted pest, and are likely to be inefficient in the suppression of pests [24]. A common predator, the striped lynx spider, *Oxyopes salticus* (Araneae: Oxyopidae; Hentz) likely predates many species in soybeans, but exhibited low levels of predation on brown marmorated stink bugs, *Hyalomorpha halys* (Hemiptera: Pentatomidae; Stål) [32]. On the other hand, in some systems, such as cereal aphids in wheat, generalist predators tested positive for pests at high rates, and diverse communities provided higher levels of predation on a pest [23]. More recently, diagnostic multiplex PCR that investigated food web structure confirmed the hypothesis that habitat structure in agricultural systems can alter the diet of generalist predators [33,34,35]. Provisioning biodiversity for ecosystem services is critical, and MGCA has helped understand prey choice, as well as whether generalist predators provide complementary or redundant predation services [33].

MGCA can be used as a tool to understand the host-plant preference of agronomic crop pests. Many species of pests are highly polyphagous, feeding on a diversity of host plants. The gut content analysis of the migratory *Apolygus lucorum* (Hemiptera: Miridae; Meyer-Dür) collected from crop-free islands revealed that this herbivore feeds on 17 plant families, but predominantly feeds on cotton [27]. However, *A. lucorum* showed a preference for mung beans over cotton [36], suggesting that mung beans may be a suitable trap crop in cotton systems. Crop diversity enhances arthropod populations and pest control [28], and a recent study using MGCA found some lower feeding on crops with intercropping as compared to monoculture [28].

Enzyme-linked immunosorbent assays (ELISA) are a MGCA tool that can be used to track the spatial movement of trophic resources. Hagler [37] tagged prey with specific proteins (typically vertebrate egg protein) and the gut of the focal arthropod was tested for the presence of protein-marked food items via a standardized protein-specific ELISA. For example, the dispersal of natural enemies from buckwheat to grapes provided evidence that buckwheat trophic interactions are connected to grape production [38]. In addition, in order to track predators in space and time, ELISA is useful for monitoring the effects of trap crops, as well as to identify predation at specific pest life-stages [39]. ELISA has proven to be a valuable tool for tracking the movement of predators and prey, and is very cost effective [37].

### 3.2. Current Application of MGCA to Understand Tree Fruit and Tree Nut Systems

The use of MGCA to reveal insights into the structure of tree fruit systems has only recently been employed. Generally, few studies have used MGCA to target perennial systems. This presents a unique opportunity, as perennial systems are complex, and some systems are slow to reach productive stages. Therefore, developing resilient and stable biological control communities early in their establishment could be an effective means of achieving long-term biological control in fruit and tree nut systems.

The key predators of targeted pests in tree fruit systems have been identified by MGCA. For example, natural predators for the suppression of a fruit boring lepidopteran pest in South China were documented with MGCA [40]. The diagnostic PCR of pest-specific primers identified Coccinellidae and Araneae as the main source of predation [40]. In Washington apple orchards, a combination of spiders, carabids, and earwigs were identified as the main predators of the codling moth *Cydia pomonella* (Lepidoptera, Tortricidae; Linnaeus). This information was used to select insecticides that were the least harmful to beneficial predators. In Michigan organic apples, species-specific primers showed that predators fed on the common apple pest plum curculio (Curculionidae: *Conotrachelus nenuphar*; Herbst), but the interaction is infrequent [41]. Recent efforts to improve biological control in Hawaiian macadamia orchards determined that ladybeetles (Coleoptera: Coccinellidae) are key biocontrol agents of the macadamia felted coccid, *Eriococcus ironsidei* (Hemiptera: Erioccocidae; Williams). The MGCA of five commonly-observed ladybeetles revealed that ladybeetles represent an essential component of the natural biological control of the macadamia felted coccid [42].

Beyond the analysis of one pest, characterizing multiple prey sources has led to a better understanding of complex interactions between pest species and predators in tree fruit systems. Parasitoids have been identified as the primary biocontrol agent of scale insects in citrus production systems. However, MCGA revealed that first generation scales were predominately consumed by Miridae and Coccinellidae, which are often documented as aphid predators [43]. As aphid populations declined, first generation scales emerged, providing evidence of density-driven prey switching [43]. Similarly, MGCA showed that spiders were important predators of early season moths in apple, but Carabids provided late-season control of diapausing larvae on the ground [44].

Another recent application of MGCA in perennial nut systems revealed biocontrol agents for the insect vector of the plant pathogenic bacterium *Xylella fastidiosa* (Lantero et al., 2018). *X. fastidiosa* is primarily transmitted by insect vectors (some species of planthoppers; piercing-sucking Hemiptera), and it causes diseases of economic importance, such as almond, and pecan leaf scorch that can severely reduce the nut yield (Rebek, 2017). MGCA identified several predators which are important in the biocontrol of *X. fastidiosa* vectors, providing evidence of predator-herbivore-pathogen interactions that may help prevent the spread of disease in almond systems.

### 3.3. MGCA to Understand Predation in Specialty Row Crops 

A limited number of studies have used molecular gut content analysis to understand predatory behavior in vegetable production. Those conducted in vegetables have explored temporal differences in prey consumption [18], documented patterns of predator movement from non-crop to crop vegetation [45], and tested the effects of farm management practices on predator abundance and prey consumption [46]. In this section, we summarize the key findings from the application of MGCA to understand vegetable production food webs.

Vegetable systems are often challenging for pests and their associated diseases, but recent studies show promise to better understand trophic interactions, and possibly to improve future sustainability. Two major pests of Mediterranean lettuce are *Nasonovia ribisnigri* (Hemiptera: Aphididae; Mosley) and *Frankliniella occidentalis* (Thysanoptera: Thripidae; Pergande); both are consumed by multiple native *Orius* species [47]. The diagnostic PCR analysis of predators for these pests and a common alternative prey, Collembola, showed the high occurrence of predation on *N. ribisnigri* and *F. occidentalis*. The patterns of predation in this system were seasonal; a greater number of *N. ribisnigri* were consumed in spring, whereas more *F. occidentalis* were consumed in summer, which follows the natural population emergence of the two species. In addition, next-generation sequencing showed that a diversity of alternative prey was consumed by these predators [47]. For biological control to function appropriately, alternative prey can help sustain predators, but at times, additional resources may distract predators from target prey. MGCA showed that two predators—*Hippodamia variegate* (Coleoptera: Coccinellidae; Goeze) and *Micromus tasmaniae* (Neuroptera: Hemerobiidae; Walker)—in *Brassica oleracea* farms move between non-crop vegetation and cropping fields, suggesting that alternative resources may benefit the biological control of some pests [45].

### 3.4. Applying MGCA to Agricultural Landscapes

To date, landscape-scale studies have typically relied on the indirect measurement of pest control services [48]. For example, sentinel eggs or nymphs established within predator exclusion cages are often used to understand the impact of natural enemies on herbivore control and population growth [49]. Although such measurements provide valuable information regarding the overall impact of natural enemies on a particular insect pest, we know little about the role that the observed natural enemies play in agroecosystems. MGCA can be used to investigate complex interactions in the agricultural landscapes, such as to track parasitism [50], predation frequency [51,52], and complex trophic food webs [51,53]. Interactions between natural enemies and pests can be linked to the degree of agricultural landscape complexity (e.g., the proportion of non-crop habitat in the surrounding landscape). In this section, we summarize developments in the application of MGCA to understand food webs in agricultural landscapes.

Estimating the rate of parasitism in agricultural landscapes via molecular analysis is an emerging frontier. In order to estimate rates of parasitism, pest tissues (i.e., egg, nymph, or adult) are collected from the environment and sequenced in order to identify and measure the frequency of parasitoid occurrence [10]. This is a particularly powerful approach for landscape-scale studies that require a large number of samples. For example, landscape structure was an important factor explaining *Lygus lineolaris* (Hemiptera: Miridae; Palisot de Beauvois) parasitism in strawberries; the parasitism rates increased as the proportion of semi-natural habitats increased in the surrounding landscape [50]. Furthermore, they observed a negative association between *L. lineolaris* parasitism and the proportion of crops in the landscape, which indicates the importance of landscape structure in determining species interactions [50].

Trophic interactions may be driven by both landscape composition and the within-field position, with multiple outcomes on biodiversity [54]. Predation on two asparagus pests was rare, but occurred more frequently on the edge of fields, and in forested margins [55]. This finding is consistent with prior work on the interaction between parasitoids and asparagus pests [56]. Both local and landscape complexity can affect the abundance of generalist predators and the activity of a polyphagous pest. In blueberry fields, local management practices did not affect *Drosophila suzukii* (Diptera: Drosophilidae; Matsumura) abundance and activity, and metabarcoding confirmed that a diversity of predators feed on a wide variety of prey with low specialization on blueberry pests, but only a few individuals (7 out of 1600) tested positive for *D. suzukii* DNA [57].

The combined analysis of parasitoids within hosts and predator gut content allows researchers to uncouple the positive interactions of predation and parasitism as well as intraguild predation in relation to the landscape contexts of cropping systems. For example, natural enemy assemblages vary temporally as a function of landscape complexity, which likely has implications for their roles in biological control. In wheat, multiplex and singleplex PCR showed that the rates of parasitism during the early season on *Sitobion avenae* (Hemiptera: Aphididae; Fabricius) were higher in simple landscapes that had high agricultural intensification [58]. On the other hand, in complex landscapes with low agricultural intensification, lady beetles contributed significantly to *S. avenae* suppression early in the season, but disrupted parasitism [53]. Studies that employ MGCA in agricultural landscapes indicate the advantage of MGCA to reveal the complex interactions between natural enemies and herbivores resulting from land-use agricultural mosaics.

### 3.5. Using MGCA to Expose Intraguild Predation

Characterizing predator–prey or parasitoid-host interactions is essential to achieve sustainable pest management strategies; however, interactions among natural enemies that utilize similar resources (e.g., predator–predator or predator-parasitoid) are also relevant to understand the underlying mechanisms behind biological control efficiency. Generalist predators likely engage in cannibalism (i.e., prey upon their conspecifics) and/or intraguild predation (by preying on heterospecific species), which can directly interfere with the abundance and distribution of the species involved, and consequently in pest management. The extent to which intraguild predation (hereafter IGP) can disrupt biological control efficiency, in many cases, is still unknown. This section focuses on MGCA-related techniques that reveal IGP and the influence of IGP on pest suppression by natural enemies.

The recognition of IGP events and their complexity should be evaluated when shaping biocontrol programs. For example, MGCA uncovered a greater-than-previously-expected likelihood of the invasive harlequin ladybeetle, *Harmonia axyridis,* preying upon native ladybeetles compared to lacewing species (Neuroptera: Chrysopidae) [58]. Interestingly, no trace of *Chrysoperla* individuals were found in the gut content of field-collected *H. axyrids.* Thus, it is reasonable to infer that, for biological control, *Chrysoperla* species may have fewer negative impacts on native predators than the invasive *H. axyridis*. In contrast, the level of IGP between similar coccinellid species in other systems was low [59]. The winter-active spider species present in pear orchards engage in IGP events; however, the frequency of the IGP observed was low compared to their consumption of pests (pear psyllids). Therefore, at least in this case, the negative disservices of IGP were counterbalanced by their efficacy of biological control on a target pest [60]. In a recent landscape study, IGP was found to be low, and did not impact predation on aphids, which supports the natural enemy hypothesis, i.e., that greater diversity of natural enemies leads to more effective biological control [53]. MGCA can also be used to determine the balance of services and disservices by evaluating predation on other beneficial insects in response to habitat management. For example, flower-associated ambush predators feed upon a wide assortment of insects, and were also found to predate bees and other natural enemies [61]. Therefore, depending on the needs of the agroecosystem and the local community, the use of flowering habitats (which is usually associated with providing resources to beneficial arthropods) could be unfavorable, or could require the careful planting of compatible flowering species, a result that can be elucidated with MGCA.

### 3.6. Roles of Vertebrates in Biocontrol Determined by MGCA

The molecular gut content analysis of vertebrates, particularly of insectivorous birds and bats, shows promising evidence that these groups may provide important ecosystem services in a variety of agroecosystems reviewed by [62]. A total of eight bat studies and four bird studies were conducted between 2015 and 2020 in agricultural systems using the MGCA approach. Commonly in vertebrate systems, fecal samples or pellets are collected for DNA analysis either by trapping individual birds or bats within the crop system of interest, or by passively collecting fecal pellets from bat roosting sites. The analysis of vertebrate pest consumption ranges from targeted single prey species [63,64] to DNA metabarcoding analysis that captures diet breadth [65,66,67], as well as the targeted sampling of a single predator [68,69,70,71] or multiple predator species or communities. Although more research is warranted, some general patterns of vertebrate pest consumption have emerged, including evidence of density-dependent foraging by birds and bats, as well as evidence of both services and disservices.

The research to date suggests that bats provide more services than disservices. The diet of some bat species shifts seasonally or as prey abundance changes, suggesting that bats may flexibly adjust their foraging patterns and diet to exploit pest outbreaks [63,69,70,71]. For example, as the pink bollworm (*Pectinophora gossypiella*; Lepidoptera: Gelechiidae; Saunders) abundance increased in cotton, the likelihood that pink bollworm was detected in Kuhl’s pipistrelle (*Pipistrellus kuhlii*) diets increased as well [70]. Similarly, bat activity and the consumption of the pecan nut casebearer, *Acrobasis nuxvorella* (Lepidoptera: Pyralidae; Neunzig) coincided with peak outbreaks in orchards [63]. The consumption of the four main pest species by insectivorous bats in macadamia was also correlated with pest outbreaks [72]. In vineyards, lesser horseshoe bats eat a variety of pest species, and their diet changes seasonally [69]. Furthermore, bats have been shown to consume not only herbivorous pests, but also pests that vector diseases. Fecal samples from six common bat species in Madagascan rice fields showed that bats foraged on economically-important pests as well as arthropods that are vectors of disease in rice [67]. A continental-wide landscape analysis across southern Europe and DNA metabarcoding showed that the dietary patterns of the common bent-wing bat are predicted by the agricultural intensification in the landscape [68].

For birds, the balance between services and disservices may depend on the crop type. Molecular gut content analysis in combination with exclusion experiments demonstrated that prairie birds lead to net services in corn, but disservices in soy fields [66]. The diets of adult and nestling western bluebirds (*Sialia mexicana*) differ; however, both provide net services in California vineyards [71]. In macadamia orchards, the analysis of eleven bird species showed overlap in diet diversity, including the consumption of five major insect pest species [65]. A DNA-based approach was used to detect whether birds were foraging on codling moths in organic apple orchards, but the detection was extremely low; only one species, the brown-headed cowbird, was found to eat codling moths (Mangan et al. 2018).

## 4. Conclusions

At a point in time at which maximizing ecosystem service delivery is essential for food security [73], MGCA is proving to be a useful tool for understanding arthropod communities. Clarifying trophic interactions in agroecosystems is critical to building successful and sustainable IPM strategies by understanding how management alters the structure and function of communities. Overall, MGCA has increased the opportunity for and broadened the scope of research on complex trophic interactions in agricultural systems. MGCA enables the identification of predation, parasitism, IGP, host use by herbivores, non-prey resources used by natural enemies, and vertebrate roles in pest management, which can be applied in massively parallel high-throughput processing to unravel thousands of interactions. This technique enables researchers to estimate the importance of natural enemies for several pest species simultaneously, and it provides a comprehensive view of natural enemies’ contributions to pest control services. Traditional taxonomic surveys are time-consuming and depend on highly specialized knowledge of all of the species which make up a community. Sequencing and MGCA requires less specialized training to enable the rapid analysis of species presence, as well as trophic linkages within the community [74]. Furthermore, the ways in which organisms interact in dynamic space and time will aid future concepts that seek to develop sustainable pest management solutions based on functionally-relevant food webs for biological control, and can help clarify the structure and function of complex agroecosystems.

In this review, we showed that research is just beginning to realize the full capacity of MGCA to understand agroecosystem food webs. For example, most of the currently-published research seldom includes pest or predator abundances, and few include the abundances of all potential interacting species (Figure 3). However, exceptions do exist, including impressive examples of network analysis to model system behavior [25,33]. While MGCA provides several advantages over classic methodologies (e.g., predator exclusion and potted plant techniques), this tool lacks the quantitative rigor that clearly links predator feeding frequency to the number of pests consumed. Although the quantification of prey consumed by MGCA is currently limited, by simply characterizing interactions along with the abundance of the upper or lower portion of the food web, modeling can be used to estimate abundance. Accordingly, MGCA can provide information on predators and predation frequency with respect to pests, which is essential information to understand the function of species in agricultural systems and document whether species provide services or disservices for the production of food and fiber. The function of most species in natural and managed systems is unknown. In addition, a continued challenge is obtaining the abundances of all of the interacting species and interaction frequencies to better model ecological food webs, and the results of climate or agricultural practices [17]. Therefore, future work that combines MGCA with cage studies of the controlled numbers of interacting species will further our understanding of predation occurrence and will contribute to our understanding of pest control [19].

Currently, most of the studies uncovered in our review focus on agronomic crops (Figure 2). Future research is needed in vegetable systems, and within the larger agricultural mosaic. Working to improve the functioning of ecological communities in vegetable systems is a significant challenge. Specialty crops are high value systems that require considerable inputs to yield economic benefits [75]. We have seen multiple instances of insecticide resistance in vegetable systems (e.g., diamond back moth, *Plutella xylostella* (Lepidoptera: Plutellidae; Linnaeus), with no efficient tools currently to control them [75]. Elucidating the interactions in communities through MGCA should help to predict the outcomes of management, and to better integrate natural pest control services into these systems. At the field scale, growers worry about habitat management (i.e., the addition of wildflower plantings) that might have greater effects on promoting pests than on natural pest management services; MGCA can be used as a tool to understand responses to habitat management and track changes in trophic interactions. Furthermore, there is growing knowledge that production systems are connected over space and time, and using MGCA to track trophic interactions across these continuums is now possible. While many studies have determined the biodiversity benefits of land-use practices, the effects of landscape composition and configuration on trophic interactions, as well as the role that vertebrate predators play in biological control is now possible with these techniques. Therefore, MGCA is opening a new window into understanding the structure and function of agroecosystems with the hope of more efficiently harvesting and preserving valuable ecosystem services.

## Data Availability

The data is available in online data archives for journals and web-based search engines.
